# ViCoW: A dataset for colorization and restoration of Vietnam War imagery

**DOI:** 10.1016/j.dib.2025.111815

**Published:** 2025-06-21

**Authors:** Duc-Minh Nguyen, Tri-Nhan Nguyen, Trung-Quan Hoang, Cao Vu Bui

**Affiliations:** FPT University, Viet Nam

**Keywords:** Vietnam War, Historical image restoration, Image colorization, Digital heritage preservation, AI in history, AI for cultural heritage, Visual storytelling with AI, Grayscale-to-color image dataset

## Abstract

This dataset presents a curated collection of 1896 high-resolution image pairs extracted from four historically significant Vietnamese films set during the Vietnam War era. Each pair consists of an original color frame and its corresponding grayscale version, generated using the ITU-R BT.601 luminance formula. Designed to support research in historical image restoration and colorization, the dataset serves as a benchmark for evaluating AI-driven colorization techniques. Frames were systematically extracted at 3 s intervals from well-preserved archival footage, followed by manual selection to ensure visual diversity and contextual relevance. The dataset is organized into training, validation, and test sets, enabling researchers to train and assess deep learning models for restoring and colorizing historical imagery. In addition to addressing the challenges posed by aged film quality, temporal degradation, and complex visual content, this dataset contributes to digital heritage preservation by making grayscale historical visuals more accessible and engaging for modern audiences. Potential applications include the development of automated colorization systems, domain adaptation research, and AI-powered video restoration from static images.

Specifications Table

**Table:** 

Subject	Computer Sciences
Specific subject area	Historical image processing, image colorization, computer vision, and deep learning.
Type of data	1896 grayscale images 1896 color images 3 tables
Data collection	The dataset was created by systematically extracting frames from archival Vietnam War-era films using OpenCV. Frame sampling occurred at 3 s intervals to capture diverse visual content across different scenes. A manual screening process then filtered out low-quality or redundant frames. The corresponding grayscale images were generated from the color frames using the ITU-R BT.601 luminance formula. All images were standardized to a resolution of 1280×720 pixels to ensure consistency.
Data source location	The source material comprises four historically significant Vietnamese films depicting events from the Vietnam War era. These films were selected for their cultural relevance and preserved image quality, enabling high-resolution frame extraction. The dataset was built using these videos, which were processed locally using Python and OpenCV. Full details about the films, including titles and production years, are provided in the main text.
Data accessibility	Direct URL to data: https://www.kaggle.com/datasets/rhythmgc/vietnamfilmframe https://data.mendeley.com/datasets/m5zmrr564v/1
Related research article	none

## 1 Value of the Data

• ViCoW is the first publicly available dataset containing images extracted from 20th-century Vietnam War-era films, offering a unique resource for studying historical visual content and supporting efforts in digital heritage preservation.
• The dataset serves as a valuable resource for developing and benchmarking AI-based image restoration and colorization models, especially those tailored to archival footage with typical degradations such as low resolution, film grain, and noise artifacts from aging and storage. Researchers can explore domain adaptation techniques by combining ViCoW with other historical or grayscale image datasets, facilitating experiments on generalization and transfer learning across temporal and cultural domains.
• The visually diverse scenes—ranging from urban battles to rural landscapes—expose models to varying lighting conditions, environmental textures, and historical objects, enriching the training and testing process for restoration algorithms.
• Image sequences in the dataset can also support research in video restoration, animation, and frame interpolation by providing realistic static inputs derived from historical footage.

## 2 Background

Restoring and colorizing old black-and-white images is an established task in image processing and digital heritage preservation [1,2]. This work focuses on historical imagery from the Vietnam War, where most photographs and film footage were captured using analog equipment. These sources typically lack color information, which presents challenges for image processing techniques.

Recent advancements in artificial intelligence have significantly improved the performance of automatic image colorization [[3], [4], [5]]. Models based on convolutional neural networks [6] and generative adversarial networks [7] have shown promising results when applied to grayscale imagery. According to a recent 2025 survey [8], commonly used datasets for image colorization include Places205 [9], ImageNet ILSVRC2012 [10], and COCO-Stuff [11]. Additionally, several recent datasets have been developed for specialized domains, such as fruit [8], historical aerial imagery [12], manga [13], underwater scenes [14], and thermal infrared images [15]. These datasets differ significantly from historical wartime visuals, which often contain domain-specific elements such as tropical forests, military equipment, and aged textures [16].

To address these limitations, we compiled the Vietnam War Image Dataset for Historical Colorization and Restoration. This data supports the development and evaluation of AI-based colorization and restoration techniques tailored to historical content.

## 3 Data Description

The dataset consists of 1896 grayscale images in Joint Photographic Experts Group (JPG) format with a resolution of 1280×720 pixels. Each image is provided in two versions:

• Color images, which serve as the ground truth.
• Grayscale images, generated from the color versions using the ITU-R BT.601 formula.

The dataset includes images from four historical films, with the following distribution:

• The Legend Makers [17]: 700 images
• Down South, Up North [18]: 535 images
• The Scent of Burning Grass [19]: 225 images
• Hanoi 12 Days and Nights [20]: 436 images

**Fig. 1:**
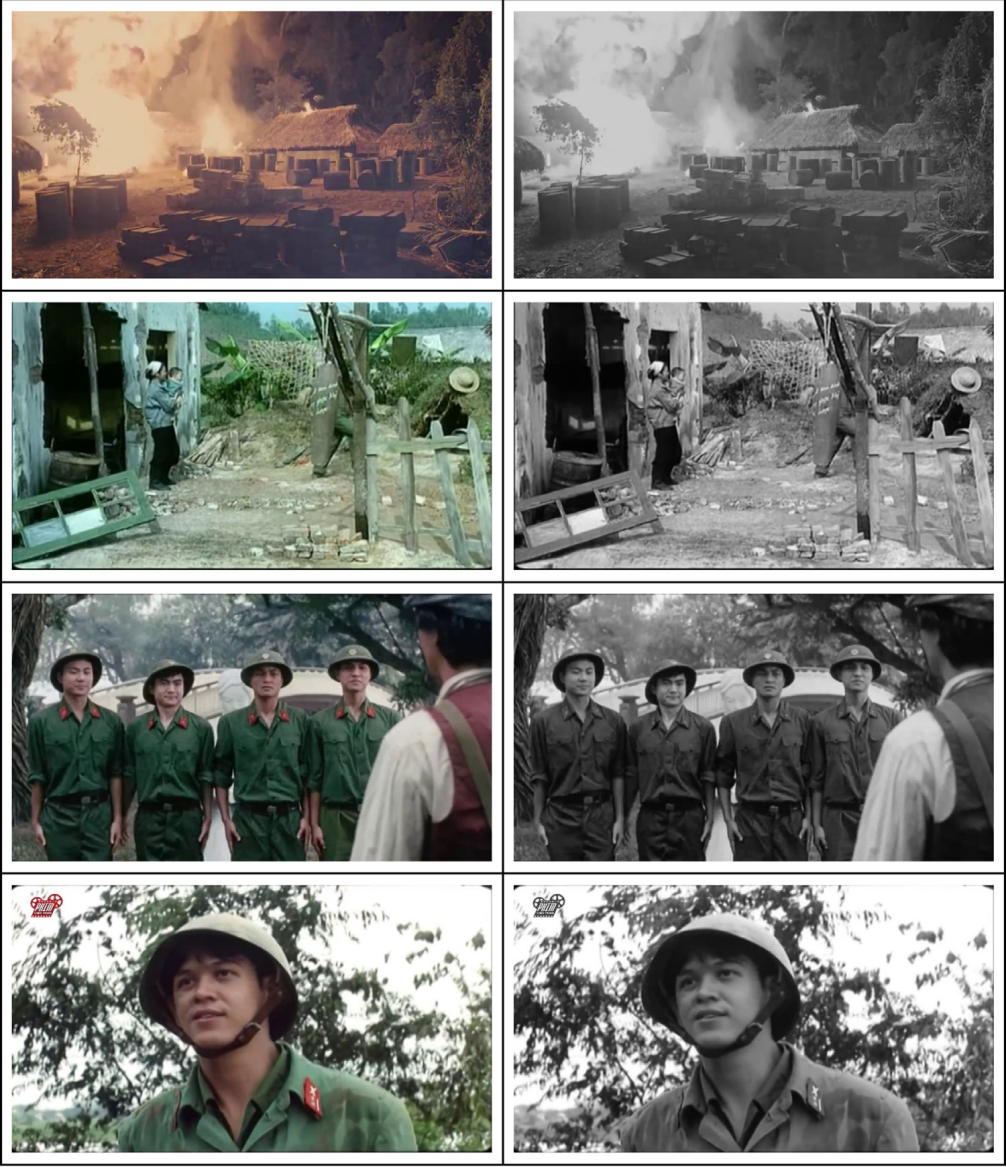
Example of image pairs from the dataset – original color frame (left) and grayscale version (right).

This dataset is designed to support research in image colorization, historical image restoration, and AI-driven digital heritage preservation Fig. 1.

## 4 Experimental Design, Materials and Methods

The dataset is built from historical images extracted from 4 Vietnam historical films. The selected sources meet the following criteria:

• Authenticity: The films illustrate real events from the Vietnam War.
• Historical significance: They have been widely broadcast and hold cultural and historical value.
• Image quality: The footage is well-preserved, allowing high-quality frame extraction.

The dataset includes images from the films shown in Table 1.

**Table 1:** Films where images (frames) are extracted from.

Film	Production year	Director	Description
The Legend Makers	2013	Bui Tuan Dung	Set in the 60s during the war, Vietnamese soldiers have to overcome numerous hardships and dangers to build an oil pipeline all the way from the north to supply the fighters in the South. [17]
Down South, Up North	2000	Phi Tien Son	A flashback that includes American bombing of a village during the Vietnam war is bookended with scenes at a nightclub in Ho Chi Minh City where a businessman thinks he recognizes a hostess as the little girl who helped him during the War when he deserted the Vietnamese Communist army. [18]
The Scent of Burning Grass	2011	Huu Muoi Nguyen	Four North Vietnamese soldiers fighting in an 81-day battle in 1972. [19]
Hanoi 12 Days and Nights	2002	Dinh Hac Bui, Trung Hai Bui	The Vietnam War from the North's viewpoint. Air raids on Hanoi stiffen the resolve of the population to strike back against a far more powerful enemy. [20]

To create the dataset, frames were extracted from the original videos at 3 s intervals. This ensures a diverse set of images while avoiding redundant frames.

The extraction process was performed using OpenCV, a widely used computer vision library. The implemented script follows these steps:

1. **Open the video file**: The program loads a video using OpenCV’s VideoCapture function.
2. **Retrieve video properties**: The script extracts the frame rate (FPS), total number of frames, and video duration. This information helps calculate the correct interval for frame extraction.
3. **Determine frame selection intervals**: Every 3 s, a frame is selected for extraction. The script calculates how many frames correspond to this time gap based on the video’s FPS.
4. **Save extracted frames**: The program saves the selected frames as JPG images in a designated output folder.

This automated process ensures that the dataset contains a diverse set of images from multiple sources while maintaining consistency in frame selection.

After automatic extraction, a manual selection process was applied to ensure the dataset contains only relevant and diverse images. The selection criteria include:

• Avoiding redundant frames: Consecutive frames were reviewed to identify near-duplicate images. If two or more frames showed minimal differences in subject movement, composition, or background, only one was kept. Frames were considered redundant if they exhibited over 90% similarity, as judged by the Structural Similarity Index Measure (SSIM). This step helped remove unnecessary repetition and ensure efficient dataset size.
• **Contextual Relevance:** Frames lacking meaningful visual elements were excluded. Reviewers ensured each image contained relevant objects or scenes, such as soldiers, military vehicles, weaponry, civilian life, or wartime settings. Frames that showed empty backgrounds, blurred transitions, or unidentifiable objects were considered irrelevant and removed to maintain the dataset’s focus and usefulness.
• Balancing scene variety: To keep many different kinds of scenes, a tagging system was used during manual checking. Each image was given one or more scene type labels, and a spreadsheet was used to track how often each type appeared. Reviewers picked frames to make sure there was variety in the scene types. Table 2 shows the types of scenes while Table 3 shows how the scene types are spread out. This step helped make sure the dataset included many real-life situations useful for training models.

**Table 2:** List of scene types.

Scene Type	Description
Combat/Battle Scenes	Images directly depicting active fighting, explosions, soldiers in engagement
Military Life (Non-Combat)	Soldiers in camps, during training, rest, daily routines, patrols (if not actively engaged)
Civilian Life/Villages	Vietnamese civilians, village scenes, market places, daily activities
Landscapes/Nature	Jungles, rice paddies, rivers, mountains without prominent human or military activity
Portraits/Individual Subjects	Close-ups of individuals (soldiers, civilians, political figures)
Logistics/Equipment	Supply lines, vehicles, weapons, machinery
Aftermath/Destruction	Damaged buildings, desolate landscapes after combat

**Table 3:** Distribution of scene types in the dataset.

Category	Film 1	Film 2	Film 3	Film 4	Total	Percentage (%)
Combat/Battle Scenes	162	10	44	16	232	12.24
Military Life (Non-Combat)	311	215	117	105	748	39.45
Civilian Life/Villages	53	157	0	100	310	16.35
Landscapes/Nature	8	2	0	3	13	0.69
Portraits/Individual Subjects	97	101	51	66	315	16.60
Logistics/Equipment	46	14	4	59	123	6.49
Aftermath/Destruction	23	36	9	87	155	8.18

This manual filtering step improves dataset quality, ensuring that each image contributes valuable information for training and evaluating colorization models.

Each extracted color image is converted to grayscale using the ITU-R BT.601 formula:

Y=0.299R+0.587G+0.114B

Where:

• R, G, B represent the red, green, and blue color channels of the image.
• Y is the computed grayscale value.

This formula is widely used in video and image processing because it preserves luminance information in a way that matches human visual perception. The converted grayscale images serve as inputs for colorization models, while the original color images act as the ground truth for training and evaluation.

The dataset is randomly divided into training, validation, and test sets using the train_test_split function from the scikit-learn library, as shown in Table 4.

**Table 4:** Statistics of our dataset split.

Split	Number of images	Percentage (%)
Train	1327	70.0
Validation	187	9.9
Test	382	20.1

The dataset was split into training, validation, and test sets in two stages.

First, 70% of the data was allocated for training, while the remaining 30% was set aside as a temporary set for further division. This was done using train_test_split with a test_size of 0.3 and a random state of 42 to ensure reproducibility. train_df, temp_df = train_test_split(df, test_size=0.3, random_state=42)

In the second stage, the temporary set was split into validation and test sets, with one-third allocated to validation and two-thirds to testing. This was again done using train_test_split, setting test_size to 0.67 and maintaining the random state of 42 for consistency. val_df, test_df = train_test_split(temp_df, test_size=0.67, random_state=42)

## Limitations

Although this dataset provides a valuable resource for image colorization research, it has certain limitations. The images focus specifically on Vietnam War scenes, which may limit the dataset’s applicability to other historical periods or modern images. Additionally, the manual frame selection process, while improving dataset quality, may introduce subjective bias in choosing relevant frames. Future improvements could involve expanding the dataset with more diverse historical images, and refining the selection process to enhance research applications.

## Ethics Statement

This dataset was created exclusively for academic and research purposes, with the objective of supporting cultural heritage preservation and advancing artificial intelligence research in historical image restoration and colorization. All images were extracted from publicly available Vietnam War-era films that are distributed through open-access archives and platforms intended for historical and educational use. Prior to dataset compilation, the terms of service (ToS) of the original sources were reviewed to ensure compliance with scraping and redistribution policies. The web resources used did not prohibit the extraction of frames for non-commercial, educational research, and no access restrictions or licensing limitations were violated.

The copyright of the original films belongs to public archives or has entered the public domain. The dataset does not include any copyrighted material from restricted or privately owned sources. Faces that appear in the dataset are those of actors, public figures, or military personnel featured in public footage, mitigating privacy concerns. No personally identifiable information is included, and anonymization is not required under prevailing data protection guidelines.

All images were screened to exclude frames containing excessive violence, distressing content, or other ethically sensitive visuals. Researchers using this dataset are expected to handle war-related imagery responsibly and in accordance with ethical standards, avoiding any form of historical misrepresentation, glorification of violence, or harm to affected individuals or communities.

## CRediT authorship contribution statement

**Duc-Minh Nguyen:** Conceptualization, Methodology, Investigation, Writing – original draft, Writing – review & editing, Project administration. **Tri-Nhan Nguyen:** Software, Visualization. **Trung-Quan Hoang:** Software, Visualization. **Cao Vu Bui:** Writing – review & editing, Validation, Supervision.

## Data Availability

Mendeley DataViCoW: A Dataset for Colorization and Restoration of Vietnam War Imagery (Original data)

## References

[1] Farella E.M. (2022). Colorizing the past: deep learning for the automatic colorization of historical aerial images. J. Imaging.

[2] Joshi M.R. (2020). Auto-colorization of historical images using deep convolutional neural networks. Mathematics.

[3] M.A. Noaman, Image Colorization: A Survey of Methodolgies and Techniques, 2022, pp. 115-130.

[4] Liu S., Zhang X. (2012). Automatic grayscale image colorization using histogram regression. Pattern Recognit. Lett..

[5] J. Lee, E. Kim, Y. Lee, D. Kim, J. Chang, and J. Choo, "Reference-based sketch image colorization using augmented-self reference and dense semantic correspondence," *arXiv preprint arXiv:2005.05207*, 2020, doi: 10.48550/arXiv.2005.05207.

[6] F.A. Baldassarre, "Deep Koalarization: Image Colorization using CNNs and Inception-ResNet-v2," 12 2017.

[7] Nazeri K.A. (2018). Articulated Motion and Deformable Objects.

[8] Anwar S., Tahir M., Li C., Mian A., Khan F.S., Muzaffar A.W. (2025). Image colorization: a survey and dataset. Inf. Fusion.

[9] Zhou B., Lapedriza A., Xiao J., Torralba A., Oliva A. (2014). Proceedings of the Advances in Neural Information Processing Systems.

[10] Deng J., Dong W., Socher R., Li L.J., Li K., Fei-Fei L. (2009). Proceedings of the IEEE Conference on Computer Vision and Pattern Recognition.

[11] Caesar H., Uijlings J., Ferrari V. (2018). Proceedings of the IEEE Conference on Computer Vision and Pattern Recognition.

[12] Farella E.M., Malek S., Remondino F. (2022). Colorizing the past: Deep learning for the automatic colorization of historical aerial images. J. Imaging.

[13] Golyadkin M., Saraev S., Makarov I. (2025). Advancing sequential manga colorization for AR through data synthesis. IEEE Access.

[14] Zhang W., Li X., Huang Y., Xu S., Tang J., Hu H. (2025). Underwater image enhancement via frequency and spatial domains fusion. Opt. Lasers Eng..

[15] Zhan W., Shi M., Chen Y., Zhang J., Zhang C., Han D. (2025). Enhancing thermal infrared image colorization through reference-driven and contrastive learning approaches. Infrared Phys. Technol..

[16] Rouquet C. (2019). Shaping the notion of media influence: the remediated images of the Vietnam War. Leaves.

[17] "The Legend Makers (2013)," Amazon, [Online]. Available: https://www.imdb.com/title/tt5004784/.

[18] "Down South, Up North (2000)," Amazon, [Online]. Available: https://www.imdb.com/title/tt0300606/.

[19] "Mui co chay (2011)," Amazon, [Online]. Available: https://www.imdb.com/title/tt2424534/.

[20] "Hanoi: 12 ngay dem (2002)," Amazon, [Online]. Available: https://www.imdb.com/title/tt2819812/.

